# Second-line chemotherapy in advanced biliary cancer progressed to first-line platinum-gemcitabine combination: a multicenter survey and pooled analysis with published data

**DOI:** 10.1186/s13046-015-0267-x

**Published:** 2015-12-23

**Authors:** Lorenzo Fornaro, Caterina Vivaldi, Stefano Cereda, Francesco Leone, Giuseppe Aprile, Sara Lonardi, Nicola Silvestris, Daniele Santini, Michele Milella, Chiara Caparello, Gianna Musettini, Giulia Pasquini, Alfredo Falcone, Giovanni Brandi, Isabella Sperduti, Enrico Vasile, on behalf of the GICO group (Gruppo Italiano COlangiocarcinoma)

**Affiliations:** Unit of Medical Oncology 2, Azienda Ospedaliero-Universitaria Pisana, Via Roma 67, 56126 Pisa, Italy; Department of Medical Oncology, San Raffaele Scientific Institute, Via Olgettina 60, 20132 Milan, Italy; Unit of Medical Oncology, Institute for Cancer Research and Treatment IRCCS, Strada provinciale 142, 10060 Candiolo, Italy; Department of Oncology, University and General Hospital, P.le S. Maria della Misericordia 15, 33100 Udine, Italy; Unit of Medical Oncology 1, Istituto Oncologico Veneto, IRCCS, Via Gattamelata 64, 35138 Padova, Italy; Unit of Medical Oncology, National Cancer Institute Giovanni Paolo II, V.le Orazio Flacco 65, 70124 Bari, Italy; Department of Medical Oncology, University Campus Bio-Medico, via Alvaro del Portillo 21, 00128 Rome, Italy; Medical Oncology A, Regina Elena National Cancer Institute, Via E. Chianesi 53, 00144 Rome, Italy; Unit of Medical Oncology, Sant’Orsola Malpighi Hospital, University of Bologna, Via Albertoni 15, 40138 Bologna, Italy; Biostatistical Unit, Regina Elena National Cancer Institute, Via E. Chianesi 53, 00144 Rome, Italy

**Keywords:** Biliary tract cancer, Cisplatin, Gemcitabine, Oxaliplatin, Second-line chemotherapy

## Abstract

**Background:**

After progression to a standard first-line platinum and gemcitabine combination (GP), there is no established second-line therapy for patients with advanced biliary tract cancers (aBTC). Indeed, literature data suggest limited activity of most second-line agents evaluated so far.

**Methods:**

We collected a large retrospective series of aBTC patients treated with second-line chemotherapy after progression to a first-line GP regimen at different Italian institutions. We then pooled the data with those reported in previous studies, which were identified with a Medline search and the on-line abstract datasets of major international oncology meetings.

**Results:**

A total of 174 patients were included in the multicenter survey: response rate (RR) with second-line chemotherapy was low (3.4 %), with median PFS and OS of 3.0 months and 6.6 months, respectively. At multivariate analysis, preserved performance status, low CA19.9 levels and absence of distant metastases were favorable prognostic factors. Data from other five presented or published series were identified, for a total of 499 patients included in the pooled analysis. The results confirmed marginal activity of second-line chemotherapy (RR: 10.2 %), with limited efficacy in unselected patient populations (median PFS: 3.1 months; median OS: 6.3 months).

**Conclusions:**

The current analysis highlights the limited value of second-line chemotherapy after a first-line GP combination in aBTC. While waiting for effective biologic agents in this setting, ongoing randomized trials will identify the optimal second-line chemotherapy regimen and validate prognostic factors for individual patient management.

## Background

Biliary tract cancers (BTC) represent an uncommon group of malignancies that includes intra- and extra-hepatic cholangiocarcinoma, tumors of the gallbladder and tumors of the ampulla of Vater [1]. The only potentially curative approach to BTC is represented by radical resection in early stage: however, surgery is burdened by a high rate of recurrence [1]. In the majority of cases, disease occurs in an advanced stage and prognosis remains poor, with median overall survival (OS) times rarely exceeding the range 10–12 months [2]. In this setting, chemotherapy constitutes the mainstay of treatment strategy. In the past decades some phase III studies demonstrated that chemotherapy improves both OS and quality of life in advanced BTC (aBTC), although the magnitude of benefit from palliative therapy is limited [3–5].

More recently, two randomized phase II and III studies have demonstrated significant survival advantage for the combination of gemcitabine and cisplatin over gemcitabine alone for patients with aBTC [6, 7]: gemcitabine plus cisplatin thus represents the current standard of care as first-line therapy. Even in the absence of phase III data formally supporting the equivalence of different platinum salts in aBTC, oxaliplatin is widely considered a reasonable alternative to cisplatin: therefore, the combination of gemcitabine and oxaliplatin is often used in clinical practice as well as clinical trials as chemotherapy backbone for the evaluation of targeted agents [2]. After progression on a gemcitabine plus a platinum derivative (GP), the value of second-line chemotherapy remains an unresolved issue. Several studies suggested that second-line treatment could be helpful for selected patients with good performance status, but no consensus has ever defined the most suitable regimen to use and the right patient to treat [8].

Lamarca et al. has recently conducted a systematic review of the literature to evaluate the level of evidence behind the use of second-line chemotherapy in aBTC patients [9]. Data from twenty-five studies (for a total of 761 patients) were collected, with a mean OS of 7.2 months, a mean progression-free survival (PFS) of 3.2 months and a response rate (RR) of 7.7 %. According to these results, the authors concluded that there is insufficient evidence to recommend a second-line chemotherapy in the whole population of patients with aBTC, although a cohort of selected cases might benefit from treatment. We have recently collected the largest series of aBTC patients treated with second-line chemotherapy [10, 11]: the results of our retrospective analysis are consistent with those reported by Lamarca and colleagues.

With regards to first-line therapy, both reports from Lamarca and our group presented high heterogeneity in terms of first-line treatments [9, 10]. Moreover, in some cases, second-line treatment was represented by targeted agents, which have not demonstrated definitive efficacy in this disease [8, 11]. We may conclude that in heterogeneous populations, second-line treatment has a fairly limited role, but a rigorous estimate of second-line treatment benefit after first-line GP is currently lacking [9, 10].

In this report we aimed to define the results of second-line chemotherapy after a first-line GP combination in a large, retrospective aBTC patients’ cohort. Moreover, we performed a systematic review of the literature and pooled our data with those of other similar studies, in order to better assess the role of second-line treatment in patients with aBTC after the failure of first-line GP chemotherapy.

## Methods

### Patients selection (retrospective analysis)

For the multicenter survey, we retrospectively identified patients with aBTC treated with second-line chemotherapy at 10 Italian Institutions between 2004 and 2013. Details about the selection criteria applied were reported elsewhere [10]. In summary, eligible patients had to have a cytologically and/or histologically confirmed diagnosis of non-resectable, recurrent or metastatic biliary tract adenocarcinoma (intrahepatic or extrahepatic cholangiocarcinoma, gallbladder and ampullary carcinoma) and a radiologically confirmed progression after first-line chemotherapy with a GP doublet (gemcitabine plus cisplatin or oxaliplatin).

### Study selection (pooled analysis)

In order to better evaluate the role of second-line chemotherapy in aBTC, we combined the patients identified in the multicenter survey with other published series of patients with similar inclusion criteria. We therefore searched for eligible studies using the Medline database. Abstracts of the proceedings of the Annual Meeting of the American Society of Clinical Oncology (ASCO), the biannual European Society of Medical Oncology Congress since 2002 (ESMO) and the annual World Gastrointestinal Congress since 2006 were also searched manually. We applied the research criteria yet described in the systematic review published by Lamarca et al. [9].

### Statistical analysis

Measure of second-line chemotherapy activity and efficacy for the retrospective cohort were the following: *i*) RR: evaluated by Response Evaluation Criteria in Solid Tumors (RECIST) v. 1.0; *ii*) PFS: measured from the date of the first cycle of second-line chemotherapy to the date of disease progression or death, whichever occurred first; *iii*) OS: measured from the date of the first cycle of second-line chemotherapy to the date of death for any cause. PFS and OS were estimated using the Kaplan-Meier product-limit method. The log-rank test was used to assess differences between subgroups.

The hazard ratio (HR) and the confidence intervals (CIs) were estimated for each variable by means of the Cox univariate model. A multivariate Cox regression model was also developed with stepwise regression (forward selection) by selecting those variables that were significant on univariate analysis. Entry and removal limits were *p* < 0.10 and *p* > 0.15, respectively.

To reduce the selection biases related to a non-randomized cohort, propensity score for the likelihood of receiving combination regimens or single-agent chemotherapy was calculated from variables unmatched [12]. By using a 1:1 nearest neighbor matching algorithm that pairs patients with the closest propensity scores within a defined limit (calipers of width equal to 0.2), the propensity score yielded 2 well-matched cohorts of 98 patients (logistic regression estimation algorithm). Multivariable Cox proportional hazard models were further performed in the final sample.

A weighted combined analysis of the data of our survey and the published data of the identified series was performed: all the available end points (RR, PFS and OS) were investigated. Median values and corresponding 95 % CIs were calculated for both PFS and OS and were weighted according to the number of patients enrolled in the analyzed studies. The ratio of the number of responding patients and the number of enrolled patients in the selected studies was used to estimate RR. Case reports were excluded from the analysis.

Statistical analyses were carried out using the statistical software package Comprehensive Meta-analysis vers. 3.3 (Biostat, Inc, Englewood, NJ, USA) and SPSS software vers. 21.0 (IBM Corporation, Armonk, NY, USA).

## Results

### Characteristics of the patients included in the multicenter survey

A total of 174 patients have been included in the retrospective multicenter survey: the clinical characteristics of this cohort are reported in Table 1.

**Table 1:** Retrospective cohort: patient characteristics

Characteristics	n. (%)
Total n. of patients	174 (100)
Sex	
Male	80 (46)
Female	94 (54)
Median age (range)	62 years (28–79)
ECOG Performance Status	
0	106 (61)
1	52 (30)
2	16 (9)
Site of primary tumor	
Intrahepatic	86 (49)
Extrahepatic	37 (21)
Gallbladder	34 (20)
Ampullary	17 (10)
CA19.9 before second-line CT	
< Median value (157 U/ml)	71 (41)
≥ Median value (157 U/ml)	83 (48)
Data missing	20 (11)
Previous response to first-line CT	
Partial response	37 (21.3)
Stable disease	70 (40.3)
Progressive disease	67 (38.4)
Median PFS to first-line CT	5.8 months
Third-line CT received	74 (43)
Platinum plus or minus gemcitabine or fluoropyrimidine	24 (14)
Irinotecan plus or minus 5-fluorouracil or capecitabine	17 (10)
Monotherapy with 5-fluorouracil or capecitabine	10 (6)
Monotherapy with paclitaxel or docetaxel	7 (4)
Monotherapy with epirubicin	5 (3)
Other regimens	11 (6)

*Abbreviations: n* number, *CT* chemotherapy, *PFS* progression-free survival; *platinum*: cisplatin, oxaliplatin or carboplatin; *other regimens*: different combinations with fluoropyrimidines and other agents (taxanes, anthracyclines, gemcitabine)

Five other trials (one phase II study [13] and four retrospective series [14–17]) reporting the results of second-line chemotherapy in aBTC patients treated with a first-line GP regimen have been identified by the research strategy.

### Results of the multicenter survey

The second-line regimens used in the 174 patients are listed in Table 2. As regards activity, RR to second-line chemotherapy was low (3.4 %; 95 % CI 0.7 %-6.1 %), even though disease control was achieved in 50 patients (28.7 %). After a median follow up of 23.0 months, 154 patients have progressed and 133 have died. Median PFS was 3.0 months (95 % CI: 2.7-3.4) and median OS was 6.6 months (95 % CI: 5.1-8.1). Seventy-four patients (42.5 %) received a third-line chemotherapy after disease progression (Table 1). No major differences in response or survival have been identified according to response and PFS to first-line therapy.

**Table 2:** Retrospective cohort: second-line chemotherapy used

Chemotherapy regimen	n. (%)
Monotherapy with 5-fluorouracil or capecitabine	49 (28)
Gemcitabine plus 5-fluorouracil or capecitabine	38 (22)
Capecitabine plus mytomicin-C	21 (12)
FOLFIRI or XELIRI	17 (10)
Retreatment with gemcitabine plus cisplatin or oxaliplatin	13 (7)
FOLFOX or XELOX	10 (6)
Epirubicin plus cisplatin plus 5-fluorouracil	9 (5)
Gemcitabine plus irinotecan	6 (3)
Monotherapy with gemcitabine	4 (2)
Other regimens	7 (4)

*Abbreviations: n* number, *FOLFIRI* 5-fluorouracil plus irinotecan, *XELIRI* capecitabine plus irinotecan, *FOLFOX* 5-fluorouracil plus of oxaliplatin, *XELOX* capecitabine plus oxaliplatin

**Fig. 1:**
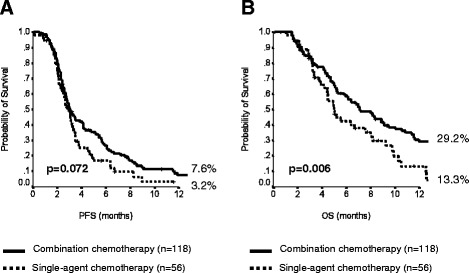
Outcome comparison between sing-agent and combination chemotherapy groups in the retrospective cohort: **a**) progression-free survival; **b**) overall survival. Legend: PFS, progression-free survival; OS, overall survival

In order to identify an optimal chemotherapeutic approach in second-line, we compared the outcome of patients receiving monotherapy with that of patients treated with a combination regimen. No imbalances in main patient characteristics (gender; age; performance status; site of origin; disease stage; previous surgery; response and PFS after first-line therapy; number of metastatic sites; presence of bone, lung, liver or peritoneal metastases; CA19.9 levels) between the two groups were identified (all *p* > 0.05). A slight increase in disease control rate (DCR) (32 % *vs.* 21 %, *p* = 0.140) and PFS (median: 3.1 *vs.* 2.9 months; *p* = 0.072) (Fig. 1a) was observed, but these differences did not reach statistical significance. Of note, we reported prolonged OS with combination chemotherapy compared to single-agent (7.1 *vs.* 5.0 months; *p* = 0.006) (Fig. 1b).

**Fig. 2:**
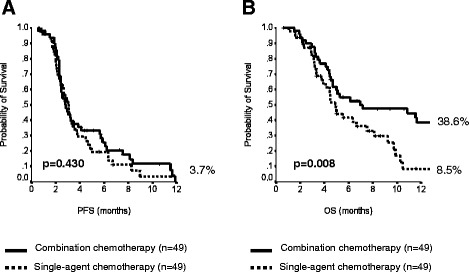
Outcome comparison adjusted for propensity score between sing-agent and combination chemotherapy groups in the retrospective cohort: **a**) progression-free survival; **b**) overall survival. Legend: PFS, progression-free survival; OS, overall survival

At multivariate analysis, the following factors resulted associated with prolonged PFS: ECOG performance status of 0, lower CA19.9 pretreatment levels, absence of lung involvement and male gender (Table 3). In terms of OS, ECOG performance status of 0, CA19.9 levels below the median value and locally advanced disease were identified as positive prognostic determinants (Table 3). When we adjusted the comparison between single-agent and combination chemotherapy by performing a propensity score analysis, the trend toward improved PFS with multi-drug regimens was lost, while the significant advantage in terms of OS in favor of the combination was actually more evident (Fig. 2).

**Table 3:** Multivariate prognostic factor analysis for PFS and OS

	HR (95 % CI)	p-value
Progression-free survival		
ECOG PS, 1/2 *vs*. 0	1.94 (1.31-2.89)	0.001
CA19.9, ≥157 U/ml *vs*. <157 U/ml	1.58 (1.08-2.33)	0.019
Gender, female *vs*. male	1.49 (1.04-2.14)	0.028
Lung metastases, yes *vs*. no	1.48 (1.02-2.14)	0.040
Overall survival		
ECOG PS, 1/2 *vs*. 0	3.56 (2.25-5.61)	<0.001
Stage, metastatic *vs*. locally advanced	3.25 (1.58-6.69)	0.001
CA19.9, ≥157 U/ml *vs*. <157 U/ml	1.96 (1.25-3.05)	0.003

*Abbreviations: ECOG PS* Eastern Cooperative Oncology Group performance status, *HR* hazard ratio; 95 % CI, 95 % confidence interval

### Results of the pooled analysis

The results of this analysis have been grouped with those of the other five trials identified [13–17], with a total of 499 patients (Table 4). All included series had data for the analysis of PFS, while only five series (for a total of 462 patients) reported OS data and four series (for a total of 420 patients) reported data on RR (Table 4).

**Table 4:** Studies included in the pooled analysis

Study [ref.]	n.	ECOG PS (n.)	Basal CA19.9 (n.)	median PFS (95 % CI) (months)	median OS (95 % CI) (months)	RR (%)
Present study	174	0: 106 1: 52 2: 16	<157 U/ml: 71 ≥157 U/ml: 83 NR: 20	3.0 (2.7-3.4)	6.6 (5.1-8.1)	3.4
He, 2014 [13]	37	0-1: 29 2: 8	normal: 9 elevated: 28	3.1 (2.3-3.6)	NR	21.6
Bridgewater, 2013 [14]	63	NR	NR	4.0 (3.3-5.6)	8.1 (5.3-11.4)	NR
Fiteni, 2014 [15]	16	NR	NR	4.0 (2.6-5.5)	5.3 (4.1-6.6)	NR
Brieau, 2015 [16]	196	0-1: 117 2–3: 56	≤400 U/ml: 81 >400 U/ml: 49	3.2 (2.8-4.0)	6.7 (5.6-7.8)	11.8
Guion-Dusserre, 2015 [17]	13	0: 3 1: 8 2: 2	median: 73 U/ml [range: 2–4472]	8 (7–16)	20 (8–48)	38.4

*Abbreviations: ref* reference number, *n* number, *ECOG PS* Eastern Cooperative Oncology Group performance status, *PFS* progression-free survival, *OS* overall survival, *CI* confidence interval, *RR* response, rate, *NR* not reported

Types of second-line chemotherapy used were the following: FOLFOX or XELOX or 5-fluorouracil plus cisplatin in 128 patients, FOLFIRI in 75 patients (in 13 of whom in combination with bevacizumab), 5-fluorouracil or capecitabine monotherapy in 39 patients, gemcitabine plus cisplatin in 17 patients and other regimens in 66 patients.

**Fig. 3:**
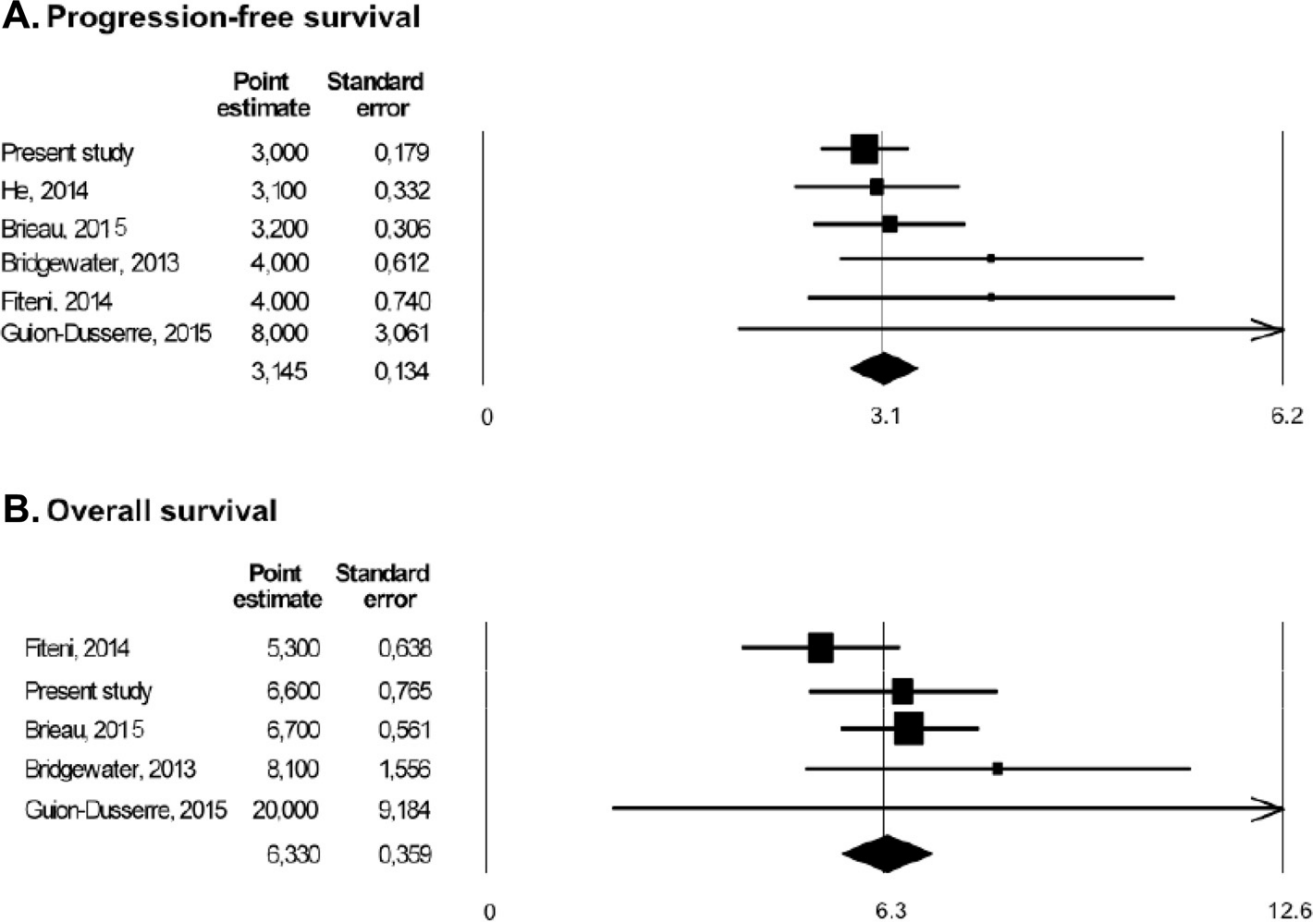
Pooled analysis of published data: **a**) progression-free survival; **b**) overall survival. Legend: Point estimates of progression-free survival and overall survival times are expressed in months

Overall RR with second-line chemotherapy was 10.2 % (95 % CI 7.3 %-13.1 %). Median PFS and OS obtained by a weighted pooled analysis of the available series were 3.1 (95 % CI: 2.9-3.4) and 6.3 (95 % CI 5.6-7.0) months, respectively (Fig. 3).

## Discussion

The GP combination has been recently set as the standard treatment for fit patients with aBTC [6, 7]. After disease progression, no established alternatives are available, and the potential benefit of second-line chemotherapy is currently under investigation in the randomized phase III ABC-06 trial, comparing FOLFOX to active symptom control (ASC) [18]. Pretreated aBTC thus represents a challenging scenario due to the paucity of reliable data about the usefulness of salvage chemotherapy.

The current analysis aims at refining the estimate of activity and efficacy of second-line chemotherapy after a standard GP combination in first-line. As in the general population of pretreated aBTC [9], second-line chemotherapy after first-line GP confirms limited activity and efficacy. We observed an overall RR of 10.2 %, with median PFS and OS of 3.1 and 6.3 months, respectively. Available data thus indicate that only a minor percentage of aBTC patients may achieve an objective response after first disease progression with GP, with a limited duration of disease control [9, 10]. These results confirm the need for a prospective evaluation of the efficacy of second-line chemotherapy, in order to definitively establish the relative benefit of medical treatment over ASC alone.

Our analysis (and the already published studies in this setting) does not allow establishing a preferable second-line agent, as formal direct comparisons among the different regimens administered would suffer from several limitations. However, we confirmed a trend toward improved efficacy with second-line combination chemotherapy compared with monotherapy, as already reported in our previous report [10]. A propensity-score analysis was conducted in order to reduce the influence of potential confounding factors on the results: the survival difference between single-agent and combination chemotherapy actually increased after adjustment, retaining statistical significance. This observation may be of value as Lamarca et al. found a correlation between RR or PFS and OS [9] and at least partially supports the use of combination chemotherapy in pretreated aBTC. Outside of a clinical trial, second-line fluoropyrimidine-based regimens (as tested in the experimental arm of the ABC-06 study) could be thus considered the most suitable option, with single-agent 5-fluorouracil or capecitabine being reasonable alternatives in unfit patients [10, 11, 19]. As mentioned, selection bias as well as the limited number of patients after adjustment for other factors may have influenced the results of our analysis: of note, Brieau et al. [16] did not report significant differences in terms of PFS and OS between monotherapy and combination chemotherapy and even in the randomized Italian trial comparing second-line capecitabine with capecitabine plus mitomycin-C no additional benefit was evident in favor of the multi-drug regimen [20]. Therefore, extreme caution is needed when trying to identify an optimal schedule in this setting.

As already described in a larger patient population [10], some clinical and laboratory parameters may help selecting optimal candidates for salvage chemotherapy. We previously demonstrated that poor performance status, elevated CA19.9 level, an on-site primary tumor and shorter first-line PFS negatively affect OS in second-line. In the current retrospective series among GP-pretreated patients, we confirmed the value of performance status and CA19.9 as major determinants of prognosis at multivariate analysis.

If the ABC-06 trial will clarify the role of second-line chemotherapy, our analysis confirms that cytotoxic therapy alone does not represent a definitive solution to the aBTC problem. Up today, the role of biologic agents in this setting is not established, and results in the first- and second-line settings are limited [8]. The largely unknown biological background behind biliary tumor progression represents one of the main limitations in the development of targeted agents, reducing the chances of an adequate molecular patient selection and tailored development of newer agents [21]. As an example, cetuximab has been recently tested in combination with gemcitabine plus oxaliplatin as first-line therapy among aBTC patients stratified by *KRAS* status [22]: results of the combination, however, remained disappointing even in the *KRAS* wild-type subgroup. More intriguingly, insights into BTC biology have recently led to the identification of potential therapeutic targets [23–27]. Of note, a recent paper has revealed that 9 % of the 65 evaluated BTC cases showed *ROS1* rearrangements at genetic analysis [23]: as in non-small cell lung cancer [28], this may pave the way for the clinical evaluation of specific inhibitors in aBTC patients.

## Conclusions

To conclude, second-line chemotherapy confirmed limited efficacy after a first-line GP regimen in aBTC, both in a large retrospective patient cohort and in a pooled analysis of published and presented data. Prospective trials such as ABC-06 are eagerly awaited to better define the role of salvage therapy compared with ASC: in the meanwhile, a fluoropyrimidine and, in selected cases, a fluoropyrimidine-based combination can be offered to patients with a more favorable prognosis, as defined by clinical and laboratory variables.

## Abbreviations

aBTC: Advanced biliary tract cancer
BTC: Biliary tract cancer
GP: Gemcitabine plus platinum derivative combination chemotherapy
OS: Overall survival
PFS: Progression-free survival
RECIST: Response Evaluation Criteria in Solid Tumors
RR: Response rate
